# Resume of the Results of Dr. Friedrich’s Researches on Abdominal Typhus (Typhoid) Fever in Children

**Published:** 1858-01

**Authors:** 


### RESUME OF THE RESULTS OF DR. FRIEDRICH'S RESEARCHES ON ABDOMINAL TYPHUS (TYPHOID) FEVER IN CHILDREN.

1st. Typhoid fever in children is not an uncommon disease; it is sometimes sporadic and sometimes epidemic. 2d. It affects boys more frequently than girls. 3d. Mortality is less among children than grown persons; greater among girls than boys. 4th. What month or season of the year predisposes children to typhoid fever does not appear. This seems more influenced by local circumstances than seasons of the year. 5th. In regard to the frequency of occurrence and mortality in different periods of life, it may be said that it very seldom attacks sucking infants after two years of age; it is most frequent between six and eleven years; from the eleventh year the susceptibility decreases until after puberty. The most unfavorable time for mortality is between one and four years. * * 7th. Typhoid of children and scarlet fever are not apt to prevail at the same time to any extent; where one occurs as an epidemic the other declines, if it was before prevailing. 8th. Occasional epidemics rage among the children to a considerable degree, while adults are entirely exempt from its attacks. 9th. The anatomico-pathological changes, in children as in grown persons, bear something of a relation with the intensity of the fever. Extremely seldom, however, is there much hardness from infiltration in the glands of Peyer; the most that is generally seen in the glands is that a single follicle is or has been infiltrated, and either by resorption or rupture into the alimentary canal, has returned to its normal size without leaving a cicatrix. The bursting of these glands into the alimentary canal is of very small extent. It is also very seldom that children show eruptive inflammation of the pharynx, œsophagus, trachea, etc. 10th. It has been seen that age and sex exercise considerable influence as predisposing causes of typhus abdominalis, but distress, uncleanliness, unsubstantial nourishment, impure air and damp, dark dwellings are most injurious. Of no less moment are acclimation, sudden changes of modes of living, and the occurrences of different and new relationship, but the most powerful of all is an epidemic morbidity of constitution. Contagion is of very doubtful importance in this respect. * * * 11th. Among the most important symptoms of typhoid fever among children are those of the abdominal organs, such as tumidity of the spleen, the diarrhœa meteorism, barborigma. There are also fever, accelerated respiration and bronchial catarrh constantly present. The infrequency and trifling character of hemorrhage occurring in the early part of the disease shows how unimportant an amount of congestion or inflammation exists in the intestinal canal in the early stages of the disease. The disease does not usually begin with the chill that ordinarily ushers it in with the adult. Nervous phenomena, delirium, somnolence, etc., are frequent but not intense in their manifestation; there is seldom any embarrassment in the motor system. Among the eruptions, the rose-colored spots are the most common; scarcely ever papular, late miliary vesicles arise. The character and extent of the eruption seem to be entirely independent of the force and intensity of the fever. 12th. Children are generally attacked in a mild degree by typhoid fever, and, as before stated, boys more severely. The duration of the disease varies with its intensity, and may be from sixteen days to several months. In severe cases, complications are very common, and very much protract, or for a long time prevent, convalescence. Relapses seldom happen, but it may be followed by other diseases. 13th. Peculiar complications are much less frequent among children than adults; such, for instance, as parotitis, phlebitis, general, but particularly, intestinal hemorrhage. The infrequency and harmlessness of this last is doubtless owing to the mildness of the intestinal lesion. The exanthemata, as small-pox, measles, variola may protract or interrupt the stage of convalescence. 14th. The ordinary result of typhoid fever in children is rapid and complete convalescence; the more favorable, because children are commonly not subject to those dangerous or mortal sequela, by which adults are affected, as tuberculosis, gangrene, ulcerations, enlargement of the glands of Peyer or mesenteric glands. Tubercles are found in very few children dying of this disease. 15th. We must depend almost entirely on objective symptoms for a diagnosis in the abdominal typhus of children; among the most important is enlargement of the spleen, next a rosealous eruption, high temperature of the skin, a knowledge of the epidemic tendency, diarrhœa, meteorism, painfulness of the abdomen, iliocoecal gurgling, catarrhal rhouchus, head symptoms, etc. A sure diagnosis cannot always be arrived at the first few days of the disease; but, gradually as the case progresses, the characteristic symptoms make their appearance, until we are enabled to recognize the disease. 16th. The prognosis in this disease in children is generally favorable; the more favorable, of course, the milder the attack, and the more simple and uncomplicated the case. The prognosis will be influenced by the character of the reigning epidemic, the circumstances, the sex and age of the patient. Complications and sequela should have more influence in making up prognosis than simple intensity. Intensity of a particular symptom is not of so bad omen, as a somewhat less intensity, but still grave *assemblage* of several symptoms. 17th. The best plan of treatment for the most part in abdominal typhus of children is the expectant. This is according to the teachings of our experience and reflection. It should be prophilactic, dietetic, and regulated by the symptoms present. The disease does not admit of interruption, though the administration of moderate doses of calomel in the first five to eight days seem to exercise a favorable influence on its general course. Above all, the strength of the little patient should be supported as much as possible, by the early and systematic use of nourishing diet, continued perseveringly during the whole course of the malady.—*All. Med. Cent. Zeitung.*

[In translating the above, as I think, interesting article of Dr. Friedrich's, of Dresden, I have used the terms typhoid fever and abdominal typhus as synonymous, as I so understand them.—TRANSLATOR.]

